# Case report: severe bradycardia, a reversible cause of “Cardio-Renal-Cerebral Syndrome”

**DOI:** 10.1186/s12882-016-0375-7

**Published:** 2016-10-26

**Authors:** Mabel Aoun, Randa Tabbah

**Affiliations:** 1 Nephrology Department of Saint-Georges Hospital Ajaltoun and Saint-Joseph University, Beirut, Lebanon; 2 Holy Spirit University of Kaslik, Jounieh, Lebanon

**Keywords:** Cardio-Renal Syndrome, Acute kidney injury, Low cardiac output, Bradyarrhythmia, Neurological deterioration, Pacemaker, Case report

## Abstract

**Background:**

Cardio-Renal Syndromes were first classified in 2008 and divided into five subtypes. The type 1 Cardio-Renal Syndrome (CRS) is characterized by acute decompensation of heart failure leading to acute kidney injury (AKI). Bradyarrhythmia was not mentioned in the classification as a cause for low cardiac output (CO) in type 1 CRS. Besides, CRS was not previously associated with central nervous system (CNS) injury despite the fact that cardiac, renal and neurological diseases can coexist.

**Case presentation:**

We report the case of a 93-year old diabetic man who presented for obnubilation. He had a slow atrial fibrillation, was not hypotensive and was not taking any beta-blocker. He developed, simultaneously, during his hospitalization, severe bradycardia (<35 beats per minute), oligoanuria and further neurological deterioration without profound hypotension. An ECG revealed a complete atrioventricular (AV) block and all his symptoms were completely reversed after pacemaker insertion. His creatinine decreased progressively afterwards and at discharge, he was conscious, alert and well oriented.

**Conclusion:**

Our case highlights the importance of an early recognition of low cardiac output secondary to severe bradyarrhythmia and its concurrent repercussion on the kidney and the brain. This association of the CRS with CNS injury-that we called “Cardio-Renal-Cerebral Syndrome”–was successfully treated with permanent pacemaker implantation.

## Background

The Cardio-Renal Syndrome (CRS) is an entity that nephrologists are often faced to. It has been defined by Ronco et al. as a pathophysiologic disorder of the heart and kidneys whereby acute or chronic dysfunction of one organ may induce acute or chronic dysfunction of the other [1]. The Cardio-Renal Syndromes are subdivided into five different subtypes in order to allow physicians to better understand the situation and offer adequate treatment [2]. The type 1 CRS is characterized by acute worsening of heart failure leading to acute kidney injury (AKI). It is considered to be the most common type of CRS and is present in approximately 25 % of patients admitted with acute decompensated heart failure (ADHF) [3]. Bradyarrhythmia was not mentioned in the classification as a cause for low cardiac output (CO) in type 1 CRS. As well as the association of type 1 CRS with central nervous system (CNS) injury has not been previously described.

**Fig. 1:**
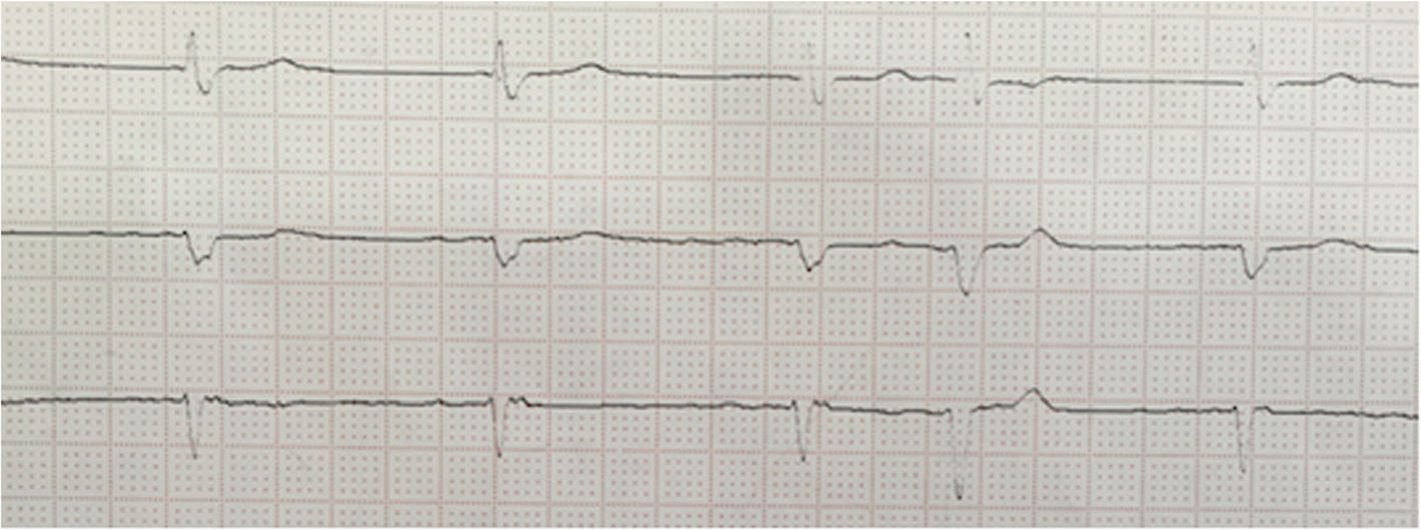
ECG on admission showing slow atrial fibrillation

We report here a case of type 1 CRS with ADHF secondary to complete atrioventricular (AV) block concomitant with neurological deterioration.

## Case presentation

A 93-year old man was brought to the emergency room for recent neurological deterioration. His past medical history was relevant for hypertension, diabetes mellitus, dyslipidemia, coronary artery disease, chronic atrial fibrillation and mild memory loss. He had no history of kidney disease. His chronic medication included clopidogrel, acenocoumarol, amlodipine, atorvastatin, molsidomine and furosemide; he was on levofloxacin since two weeks for pneumonia. On admission, the patient’s physical examination revealed obnubilation with episodes of agitation, a blood pressure of 160/80 mmHg, an irregular heart rate of 50 beats per minute (bpm), jugular venous distention, decreased bilateral basal breath sounds, no fever and no leg edema. He was not obese. Initial abnormal serum laboratory results included: white blood cell (WBC) count of 12630/mm3 (84 % neutrophils), international normalized ratio (INR) of 4.5, creatinine of 2.3 mg/dL (203 μmol/L), blood urea nitrogen (BUN) of 80 mg/dL (28.5 mmol/L), albumin of 3.2 g/dL (32 g/L), phosphorus of 5.9 mg/dL (1.96 mmol/L) and gamma glutamyl transferase of 275 UI/L. He had a positive troponin I of 0.27 ng/mL but normal CPK and CPK-MB. C-reactive protein was 19 mg/L. He had no electrolytes’ disturbance (potassium 4.9 mmol/L, sodium 144 mmol/L, chloride 110 mmol/L, bicarbonate 23 mmol/L), no hypocalcemia (corrected calcium 8.9 mg/dL) nor anemia (hemoglobin 13.4 g/dL, hematocrit 42 %). Urinalysis showed 3+ protein.

Further investigations revealed left lung basal consolidation with bilateral effusion on chest X-ray. The arterial blood gas (ABG) showed: pH 7.27, PaO2 91 mmHg, PaCO2 52 mmHg, HCO3ˉ 23 mmol/L. Electrocardiogram (ECG) on admission showed a slow atrial fibrillation of 55 bpm (Fig. 1). Brain computed tomography (CT) scanning ruled out possible ischemic or hemorrhagic stroke.

The patient was admitted to the intensive care unit (ICU). An echocardiography showed a left ventricular ejection fraction (LVEF) of 40 %, ventricular hypertrophy, pulmonary arterial pressure of 40 mmHg, dilated right ventricle and slightly enlarged left atrium. Renal ultrasound was normal.

Empiric antibiotics were started combining ceftriaxone and levofloxacin; fluconazole was added at day 2 after sputum cultures grew Candida albicans. Urine culture was sterile.

During cardiac monitoring in the ICU, episodes of extreme bradycardia were noticed as low as 32 bpm with maintained systolic blood pressure at 100–120 mmHg and very low diastolic blood pressure of 30–40 mmHg.

On day 3, the patient became unconscious and developed severe respiratory acidosis with ABG showing: pH 7.16, PaCO2 64 mmHg, PaO2 75 mmHg, HCO3ˉ 22 mmol/L and he was intubated. He concomitantly became oligoanuric and serum creatinine started to increase. A spot urine test revealed a sodium (Na) of 22 mmol/L and potassium (K) of 34 mmol/L with a ratio Na/K <1. He received sodium bicarbonate whenever his bicarbonate level was less than 22.

Complete AV block was then identified on ECG. A temporary pacemaker was implanted on day 5 and the patient was extubated. Serum creatinine decreased and urine output improved considerably. Table 1 illustrates the heart rate (HR), urine output, systolic and diastolic blood pressure values, serum creatinine and potassium variations before and after the pacemaker insertion.

**Table 1:** Daily variation of blood pressure, heart rate (HR), BUN, serum creatinine and potassium with urine output (oliguria < 400 ml/d and anuria < 100 ml/d)

	Blood pressure in mmHg (systolic/diastolic)	HR	BUN (mg/dL)	Serum creatinine (mg/dL)	Serum potassium (mmol/L)	Urine output (ml/day)
Day 1	150/55	50	80	2.3	4.9	600 ml
Day 2	130/55	40	82	2.46	5.1	600 ml
Day 3	110/45	33	89	2.77	5.5	Anuria
Day 4	110/40	34	110	3.37	5.2	Oliguria
Day 5	100/30	32	128	4.01	5.5	Oliguria
Day 6	110/45	85	133	3.74	4.7	5200 ml
Day 7	110/50	82	104	2.44	4.2	4300 ml
Day 8	105/55	82	75	1.37	3.7	1800 ml
Day 9	130/60	84	66	1.58	4.4	1700 ml
Day 10	130/60	60	55	1.5	4.2	1600 ml
Day 11	140/60	60	55	1.66	4	1250 ml

The patient finally got a permanent single chamber pacemaker VVIR and improved considerably his consciousness and renal function. At discharge he had a serum creatinine of 1.6 mg/dL and he was conscious, alert and well oriented.

## Discussion

Depending on every patient’s situation, Ronco et al. described four subtypes of type 1 CRS: 1) de novo cardiac injury leads to de novo kidney injury; 2) de novo cardiac injury leads to acute-on-chronic kidney injury; 3) acute-on-chronic cardiac decompensation leads to de novo kidney injury; and 4) acute-on-chronic cardiac decompensation leads to acute-on-chronic kidney injury [4]. Our patient can be classified in the fourth category of type 1 CRS. The severe bradycardia decompensated his chronic heart failure and led to AKI on the top of a probable chronic diabetic nephropathy. AKI in our patient was diagnosed using the definition of RIFLE, AKIN and KDIGO classifications as an increase in serum creatinine to more than 1.5-fold over baseline [5]. The factors usually implicated in the pathogenesis of AKI in type 1 CRS are multiple and complex [4]. But the main classical pathophysiological mechanism to consider first in any type 1 CRS remains the hemodynamic factor: an inadequate renal perfusion secondary to an acute low CO or acute heart failure (HF). In the classification of type 1 CRS, the authors did not emphasize on the bradyarrhythmia as a possible aetiology for low CO [1]. Patients with ADHF and type 1 CRS have usually poor outcomes especially when accompanied by severe hypotension that is not reversible [6]. But low CO, that is generally believed to occur secondary to a decreased LVEF, results sometimes from bradyarrhythmia [7, 8]. Patients with HF due to left ventricular (LV) pump failure and those with HF due to bradyarrhythmia have similar reductions in CO. However the stroke volume is reduced in patients with LV pump failure whereas it is increased in bradyarhythmia [7]. This increase in stroke volume is mandatory to ensure the hemodynamic stability of the patient with bradycardia. The inability to increase stroke volume due to decreased myocardial reserve secondary to a pre-existing heart failure decompensates the patient [8]. The fact that our patient had a previous low LVEF contributed to his hemodynamic instability once the severe bradycardia occurred. And the low urinary sodium suggested the presence of a low CO.

We found one case in the literature that combined repetitive oliguric AKI with intermittent third-degree AV block secondary to propranolol [9]. The cause of bradycardia and complete AV block in our patient was not completely clarified. No medications, no hyperkalemia and no extrinsic factors incriminated with bradycardia were found [10]. One plausible hypothesis that could explain our patient’s signs and symptoms and sequential events is the presence of a sick sinus syndrome. It is well known that, at least, 50 % of patients with sick sinus syndrome develop alternating bradycardia and tachycardia with atrial fibrillation as the tachyarrhythmia. Sick sinus syndrome is more frequent in the elderly and lead to end-organ hypoperfusion mainly cerebral hypoperfusion. And in 50 % of the cases AV block complicates the sick sinus syndrome [10, 11]. The diagnosis is challenging in most of the cases. In our case, the absence of documented sinus bradycardia make the diagnosis of sick sinus syndrome more difficult to establish. But whether the complete AV block was superimposed on a sick sinus syndrome or not, the only therapy to be considered in our symptomatic severely bradycardic patient was pacemaker placement [12]. Pacemaker therapy is indicated only to relieve symptoms in sick sinus syndrome and improve the patient’s quality of life but unfortunately has not been shown to affect survival rates [11]. The successful reversal of AKI and obnubilation after cardiac pacing confirms the fact that complete AV block was the cause behind all our patient’s symptoms.

Concerning the association of low CO with cerebral hypoperfusion, there have been several reports in the literature. First, it is known that chronic heart failure is associated with abnormal brain changes, including cognitive impairment, structural changes, and dementia [13, 14] and may have contributed to the chronic mild memory loss of our patient. On the other hand, subtle cardiac dysfunction (as quantified by cardiac output) is believed also to be related to CNS injury [15]. Bradycardia, particularly, is historically known to be correlated with vasovagal syncope but neurological deterioration may also occur without severe hypotension and collapse; a small cohort of severely bradycardic elderly patients showed that pacemaker implantation improved their mental deterioration [16]. Finally, complete AV block has also been associated with drug-resistant epilepsy [17, 18].

## Conclusion

As a summary, acute lowering of CO secondary to complete AV block can lead to renal and cerebral hypoperfusion. Physicians should be aware that ejection fraction and cardiac output are not similar, with cardiac output being more reflective of systemic blood flow. Bradyarrhythmia is a cause of decreased cardiac output and can lead to hemodynamic instability if superimposed on pre-existing heart failure, even in well preserved systolic blood pressure cases. This hemodynamic instability may cause simultaneously oligoanuria and obnubilation. We conclude that complete AV block is a cause for -what we called- a Cardio-Renal-Cerebral Syndrome. Both renal and cerebral injuries are reversible after pacemaker implantation.

## Abbreviations

ADHF: Acute decompensated heart failure
AKI: Acute kidney injury
AV: Atrioventricular
CNS: Central nervous system
CO: Cardiac output
CRS: Cardio-renal syndrome
LVEF: Left ventricular ejection fraction
